# Psychiatric Complications After Bariatric Surgery: A Narrative Review

**DOI:** 10.5152/eurasianjmed.2026.251238

**Published:** 2026-07-30

**Authors:** Tunahan Sun

**Affiliations:** 1 Department of Psychiatry, Adana Dr. Ekrem Tok Mental Health and Diseases Hospital, Adana, Türkiye

**Keywords:** Bariatric surgery, eating disorders, multidisciplinary follow-up, pharmacokinetic changes, psychiatric complications

## Abstract

Although bariatric surgery (BS) is a well-established and effective method for treating obesity, it carries the risk of developing psychiatric complications in the postoperative period. This narrative review is based on a literature search in PubMed, Scopus, and Google Scholar, covering studies published between January 2010 and September 2025. This review comprehensively examines various psychiatric conditions that may emerge following BS, including depression, anxiety, eating disorders, psychotic disorders, alcohol use disorders, and substance use disorders. The neurobiological and psychosocial mechanisms underlying these postoperative psychiatric symptoms are evaluated, and the effects of pharmacokinetic changes on psychiatric pharmacotherapy are discussed in this review. Additionally, special risk populations, psychiatric contraindications to surgery, and treatment and follow-up strategies are reviewed in light of the current literature. Multidisciplinary assessment and long-term psychiatric monitoring appear essential to optimize psychiatric outcomes and maintain the long-term success of BS.

**Main Points**

- Bariatric surgery effectively treats obesity but carries significant psychiatric risks, including depression, anxiety, eating disorders, substance use, and suicide.
- Preoperative psychiatric assessment is essential to identify contraindications such as active psychosis, severe depression, or substance use disorder. Comprehensive psychiatric screening should therefore be integrated into the routine preoperative evaluation of bariatric surgery candidates.
- Psychiatric outcomes frequently show a biphasic pattern characterized by initial improvement and subsequent relapse, underscoring the importance of long-term monitoring. Regular psychiatric follow-up is particularly important during the first 2–3 years after surgery, when psychological changes and relapse of symptoms may emerge.
- Neurobiological, hormonal, and psychosocial mechanisms may jointly contribute to postoperative psychiatric complications. Clinicians should therefore consider both biological factors (e.g., hormonal and microbiota-related changes) and psychosocial factors when evaluating postoperative psychiatric symptoms.
- Multidisciplinary care, psychoeducation, and psychotherapy (CBT, DBT) are crucial for prevention, early detection, and optimal mental health outcomes after surgery. Close collaboration between surgeons, psychiatrists, psychologists, and dietitians is essential for safe long-term management.

## Introduction

Bariatric surgery (BS) refers to a group of surgical procedures designed to promote substantial weight loss in patients with morbid obesity. The most common procedures include Roux-en-Y gastric bypass (RYGB) and sleeve gastrectomy.^1^ These operations result in significant weight loss in patients and improve obesity-related conditions, such as metabolic syndrome, cardiovascular disease, type 2 diabetes, hypertension, and sleep apnea.^2^ Additionally, individuals with chronic illnesses may experience a reduction in anxiety and depressive symptoms and improved body image after surgery.^3^ However, there are various risks of psychiatric complications following surgery. In some cases, patients’ expectations may not be fully met. In these patients, eating disorders (EDs) may persist, or eating habits may develop into substance use disorders (SUDs).^1^^,^^3^ Therefore, it is important to assess BS candidates psychologically before surgery and to conduct postoperative psychiatric follow-up.

This review examines the preoperative psychiatric profiles of patients undergoing BS and the psychiatric complications that may arise postoperatively, in light of the scientific literature. In particular, it aims to comprehensively address psychiatric complications that may occur during the postoperative period. Although several systematic and umbrella reviews have examined mental health outcomes after BS, many of these studies focus on specific psychiatric outcomes such as depression, anxiety, or alcohol use disorders. In contrast, the present narrative review aims to provide a broader clinical perspective by integrating multiple psychiatric domains, including mood and anxiety disorders, ED, SUDs, personality pathology, psychotic disorders, and suicide risk. In addition, this review discusses potential neurobiological and psychosocial mechanisms, pharmacokinetic considerations related to psychotropic medications, and psychiatric risks in special populations. By synthesizing these diverse aspects, this review aims to provide a comprehensive, clinically oriented overview of psychiatric complications associated with BS.

The main types of BS are presented in Table 1 to provide a concise overview of the surgical approaches discussed in this review.

### Literature Search Strategy

This narrative review was prepared to synthesize the current literature on psychiatric complications associated with BS, with a particular focus on preoperative psychiatric assessment, postoperative mood and anxiety symptoms, ED, alcohol and substance use disorders (ASUD), personality pathology, psychotic disorders, self-harm and suicide risk, underlying neurobiological and psychosocial mechanisms, psychopharmacological considerations, and special populations.

A literature search was conducted in PubMed, Scopus, and Google Scholar for articles published between January 2010 and September 2025. The search strategy included combinations of the following keywords and related terms: bariatric surgery, metabolic surgery, gastric bypass, Roux-en-Y gastric bypass, sleeve gastrectomy, psychiatric complications, mental health, psychiatric assessment, depression, mood disorders, anxiety, eating disorders, alcohol use disorder, substance use disorder, addiction, personality disorders, psychotic disorders, suicide, self-harm, emotion dysregulation, psychotropic medications, and pharmacokinetics.

Priority was given to human studies, including observational studies, cohort studies, systematic reviews, meta-analyses, and relevant clinical guidelines. Articles involving adult and adolescent populations were included when they contributed to the understanding of psychiatric outcomes after BS. Non-English publications, animal studies, and reports with limited relevance to psychiatric outcomes were excluded. In addition, the reference lists of relevant reviews and key articles were manually screened to identify further pertinent studies. When multiple sources addressed the same topic, greater emphasis was placed on more recent and methodologically robust evidence, particularly systematic reviews, meta-analyses, large cohort studies, and current clinical guidelines.

Given the narrative nature of the review, the aim was not to conduct a formal systematic synthesis but to provide a clinically oriented, thematically integrated overview of the psychiatric sequelae of BS and their implications for long-term multidisciplinary care. The author used ChatGPT (OpenAI, GPT-5 version) to assist in English language editing and style improvement of the manuscript. The author take full responsibility for the accuracy and integrity of the content.

### Psychiatric Evaluation Before Bariatric Surgery

The rate of psychiatric comorbidity is high among patients who are candidates for surgery. It is well-established that there is a bidirectional relationship between obesity and poor mental health. Depressive disorders, anxiety disorders, and ED are more prevalent in these individuals than in the general population.^4^ Approximately 27%-47% of patients have been reported to have diagnoses of major depression, anxiety, and sleep disorders, while 22%-24% have been diagnosed with personality disorders.^2^ In a recent study, 31% of 234 obese patients undergoing preoperative evaluation had a history of depression, and 12.8% had ED. Binge eating disorder (BED) was detected in 6.4% of patients, bulimia nervosa in 3.2%, and night eating syndrome in 3.4%. These patients exhibited abnormal eating behaviors, such as snacking, hyperphagia, emotional eating, and compulsive eating. In addition, childhood trauma was reported in 22% of patients.^5^

**Figure 1.**
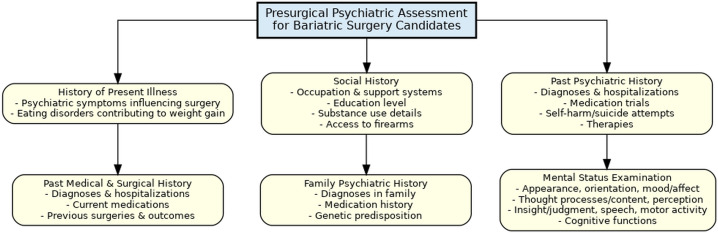
Presurgical psychiatric assessment for bariatric surgery candidates. This figure illustrates the key components of the psychiatric evaluation process conducted before bariatric surgery.

Although there is ongoing debate as to whether this psychiatric burden is a result or cause of obesity, mental health screening is critically important in BS candidates. Therefore, a detailed preoperative psychiatric evaluation is required. The primary goal of preoperative psychiatric evaluation is to identify mental health conditions that could compromise the safety of the operation or disrupt postoperative compliance. During the interview, a comprehensive assessment of the patient’s expectations for the operation, motivation, nutrition and eating habits, family or support system, educational level, occupational status, history of alcohol and substance use, past psychiatric history, medical and surgical history, and family psychiatric history should be conducted. Additionally, a thorough mental status examination should be performed to assess the patient’s capacity for adherence and engagement with postoperative follow-up, especially during the postoperative period.^1^ Bariatric surgery candidates may sometimes attempt to minimize their issues to qualify for the surgery. Therefore, an experienced psychiatrist must conduct the interview.^6^ The detailed steps of the preoperative psychiatric assessment are outlined in Figure 1.

Active ASUD, uncontrolled psychotic disorders, severe mood episodes, unstable personality pathology, and untreated severe ED may represent important psychiatric contraindications to surgery. The Italian Society of Bariatric Surgery has included a history of suicide attempts in its list of absolute contraindications for surgery.^6^^,^^7^ Some studies have suggested that certain personality traits, such as low preoperative cooperation and high impulsivity, may be associated with poor postoperative outcomes.^8^^,^^9^ Similarly, it has been reported that postoperative weight loss success may be reduced in some patients with active BED.^6^ However, the research findings on this topic are inconsistent. A recent history of psychiatric hospitalization may also necessitate delaying the operation.^7^ However, treated psychiatric disorders are not contraindications for surgery. With proper follow-up, these patients can achieve weight loss and improved quality of life.^2^ The major psychiatric conditions considered contraindications for BS are summarized in Table 2.

Preoperative psychiatric preparation involves assessing the patient’s risk and ensuring that their expectations are realistic. Patients who are candidates for BS might think that the surgery will permanently fix all their eating issues and that they will achieve normal weight without any effort. Such unrealistic expectations can lead to disappointment, depression, and adjustment disorders when the targeted weight loss is not achieved or when there is difficulty adhering to the dietary restrictions. Therefore, during the preoperative period, a multidisciplinary team comprising a general surgeon, mental health professional, and dietitian should meet with the patient to provide a detailed explanation of the surgical procedure, potential risks, necessary lifestyle changes, and the patient’s responsibilities. Providing appropriate medication, psychotherapy, or combined treatments to patients diagnosed with depression and anxiety disorders during the preoperative period can improve postoperative outcomes.^6^

### Common Psychiatric Complications Following Surgery

Patients generally experience a noticeable improvement in their mental state during the initial phase after BS (the honeymoon phase). The increased mobility, improved appearance, and reduced health problems resulting from weight loss lead to decreased depressive symptoms, greater self-confidence, and improved quality of life for most patients during the first 1-2 years. In a significant proportion of patients with depression, the need for antidepressants (AD) decreases, or depression goes into remission during the postoperative period. This pattern, however, is not observed in all patients. Evidence suggests that postoperative mental health trajectories may be biphasic, with a decline following the honeymoon phase. Over time, mental health may deteriorate in some patients.^6^

### Mood Disorders and Anxiety Disorders

Studies have demonstrated a notable reduction in depression and anxiety symptoms after surgery. Overall, there is relatively strong evidence from systematic reviews, meta-analyses, and longitudinal studies for short-term improvement in depressive and anxiety symptoms after BS, although long-term outcomes appear more variable. For example, according to the results of a recent review, depressive symptoms significantly decrease in patients who have undergone BS, and this improvement continues for at least 2 years. Similarly, a significant decrease in anxiety symptoms has been observed, particularly in women over 40 years of age.^3^ A recent meta-analysis reported that the prevalence of depression after BS is 15.3% and that depression is positively associated with weight gain and negatively associated with ED.^10^ In a recent study, 46.1% of patients experienced anxiety symptoms and 48.2% experienced depression symptoms before surgery, while 1 year later, these rates dropped to 9.5% and 22.3%, respectively.^11^ Although the incidence of depression after surgery often decreases, new depressive episodes may occur in some patients, or preexisting depression may recur. Long-term follow-ups show that depression that initially improved can worsen again in some patients after 2-3 years.^6^ For example, a 9-year follow-up study found that the lifetime prevalence of depression or bipolar disorder in patients who underwent BS increased by 32%.^12^

Overall, depression and anxiety often decline after BS. However, this effect may not be observed in every patient. Long-term psychiatric follow-up is essential for preventing late-onset recurrence. Multiple factors can contribute to the recurrence or initial onset of depression after surgery.^6^^,^^13^ Key biological and psychosocial factors associated with postoperative depression are summarized in Table 3.

Taken together, the literature suggests that BS is generally associated with short-term improvements in depressive and anxiety symptoms. However, findings across studies are not entirely consistent. While many longitudinal studies report significant psychological improvement during the first postoperative years, other studies indicate that these benefits may diminish over time or vary across patient subgroups. These differences may reflect variations in ­follow-up duration, patient characteristics, preexisting psychiatric conditions, and postoperative ­psychosocial adaptation. Therefore, although there is broad consensus that BS may improve mental health outcomes in the short term, the long-term psychiatric trajectory appears to be more heterogeneous and requires continued clinical monitoring.

### Eating Disorders

Many morbidly obese patients have ED, such as emotional eating, night eating syndrome, and bulimia nervosa, before surgery. After surgery, reduced stomach capacity together with changes in appetite-regulating hormones, including decreased ghrelin levels and increased secretion of glucagon-like peptide-1 (GLP-1) and peptide YY (PYY), may contribute to reduced appetite, enhanced satiety, and difficulty consuming large portions.^1^ As a result, many patients report a greater sense of control over their food intake. Therefore, in the early period after BS, a significant portion of these ED cases naturally improves. Several studies suggest that BED symptoms often decrease, and during the first 1-2 years, many patients do not meet the Diagnostic and Statistical Manual of Mental Disorders (DSM) diagnostic criteria. For example, Chao et al^14^ reported that only 12.5% of patients diagnosed with BED continued to meet BED criteria 2 years after surgery, and that no new BED developed in the subsequent 1-2 years in patients who did not have BED before surgery. Furthermore, a meta-­analysis reported that the incidence of ED after BS was 7.83%. This rate is lower than during the preoperative period, and BS shows a general improvement in eating behavior. In particular, the prevalence of BED decreased significantly in the postoperative period. Only 3.81% of patients met the diagnostic criteria after the surgery.^3^ However, a significant number of patients may still experience “loss of eating control” after surgery. Chao et al^14^ reported that 45.8% of patients with preoperative BED continued to report such episodes postoperatively. These findings suggest that BS may reduce binge-eating episodes, but patients with preoperative BED should be monitored more carefully.

Additionally, new or atypical forms of BED may develop postoperatively. “Bariatric binge eating disorder (Bar-BED)” has been proposed in the literature to describe binge-type eating behaviors observed after BS. Unlike DSM-5 BED, Bar-BED does not require the consumption of an objectively large amount of food because anatomical restrictions following BS often limit the quantity of food intake. Objective binge episodes required for DSM-5 BED are frequently difficult after surgery due to reduced gastric capacity; however, patients may still experience loss-of-control eating despite consuming relatively small quantities of food.^6^^,^^15^ Therefore, Bar-BED is not a formal DSM-5 diagnosis but rather a proposed clinical construct used to describe ED psychopathology in post-bariatric patients whose symptoms resemble BED despite the absence of objectively large food intake. Bariatric-BED is a poor prognostic sign because the individual will frequently eat outside of their control, which may reduce the success of weight loss. In 1 study, weight loss outcomes in patients who developed Bar-BED after sleeve gastrectomy were worse than those in patients who did not develop any ED.^15^ Therefore, patients’ eating behaviors should be thoroughly evaluated during postoperative follow-up. It should be determined whether they experience moments of loss of control, how often they snack between meals, and whether they engage in behaviors such as vomiting or laxative use.

Although rare, new-onset anorexia nervosa cases have been reported following BS. Dramatic weight loss and changes in body image can trigger restrictive eating behaviors in susceptible individuals.^16^ Additionally, some patients may develop bulimia nervosa-like behavior. Because “dumping syndrome” can occur after RYGB, especially when eating sweet or high-calorie foods, some patients might vomit to avoid this discomfort. This syndrome may eventually lead patients to avoid foods that are high in sugar and fat. Furthermore, anxiety and depression levels have been found to increase in individuals with dumping syndrome.^17^

Grazing, on the other hand, is the tendency to eat often and in small amounts after BS. A systematic review found that grazing occurs in 16.6%-46.6% of cases and may hinder the success of weight loss.^18^

Overall, the available evidence suggests that BS is generally associated with a reduction in BED in the early postoperative period. This conclusion is supported by moderate evidence. However, findings remain heterogeneous, and evidence is more limited regarding postoperative ­loss-of-control eating, grazing, and proposed constructs such as Bar-BED. While many patients show improvement in classic BED symptoms, some continue to experience loss-of-control eating or develop atypical eating patterns such as grazing. These differences may reflect variations in surgical procedures, patients’ psychological characteristics, and differences in follow-up duration. Therefore, although BS may improve eating behavior in many patients, ongoing monitoring of eating patterns remains important in the postoperative period.

### Alcohol and Substance Use Disorders and Addiction

Interest in alcohol use disorder (AUD) and SUD among patients undergoing BS has increased in recent years. Since some studies have indicated an increase in AUD after BS, particular emphasis has been placed on alcohol use.^19^ From a neurophysiological perspective, procedures such as RYGB can change the way alcohol is processed. Skipping a large part of the stomach causes alcohol to be absorbed more quickly and reach its peak level faster in the blood. Blood alcohol levels can increase by nearly 50% in individuals who consume the same amount of alcohol.^3^ As a result, patients who have undergone RYGB may become intoxicated more quickly with smaller amounts of alcohol and could be more susceptible to the rewarding effects of alcohol. Studies have also indicated that patients who undergo RYGB have a higher risk of AUD after BS than those who undergo procedures such as sleeve gastrectomy or gastric banding.^3^^,^^19^

While no significant change in AUD incidence was observed in the first 1-2 years after BS, a marked increase was reported from the third year onwards.^3^ In a study by King et al,^20^ an increase in alcohol use was reported in patients with BS within a few years after surgery, with the prevalence of AUD rising from 7% in the preoperative period to 11% after 2 years. Interestingly, some patients who did not consume alcohol before BS may begin to use alcohol problematically after surgery. This phenomenon has been proposed in the literature as “addiction transfer,” suggesting that some patients may shift reward-seeking behaviors from food to alcohol or other substances after surgery. Accordingly, some patients may turn to alcohol or other substances after surgery as alternative sources of reward when eating-related reinforcement is reduced.^1^^,^^6^ This hypothesis has not yet been fully proven, but it may be helpful for clinicians to consider this possible mechanism during the postoperative period.

There are limited data available on SUD. A retrospective study of veteran patients in the United States found a significant increase in chronic prescription opioid use after BS compared to those who did not undergo BS. Specifically, among veterans who underwent RYGB and sleeve gastrectomy, a notable increase in the incidence of chronic prescription opioid use after BS was reported within 5 years.^21^ Another study found that the prevalence of opioid use within 7 years after BS was 14.2% among patients who did not initially use opioids. In this study, opioid use decreased from 14.7% at baseline to 12.9% within 6 months but increased to 20.3% in the seventh year, surpassing the baseline level.^22^

Recurrence rates of smoking after BS are high, with studies indicating that 17% of patients continue to smoke post-surgery. Smoking has been shown to have little or no impact on weight loss after BS (maximum difference of 3%) and significantly increases the risk of perioperative and postoperative complications, such as wound healing issues, marginal ulcers, and pulmonary problems.^23^

Factors that increase the risk of substance use include a history of substance use before surgery (especially alcohol or other substances), family history of addiction, young age, male sex, and a history of past trauma.^6^ Since the type of BS (more frequent occurrence of AUD after RYGB) is also an important factor influencing this risk, other types of BS may be preferred for patients with a history of AUD. In any case, patients should be educated about ASUD after BS. Alcohol and substance use should be assessed during postoperative follow-ups, and screening scales, such as the Alcohol Use Disorders Identification Test – Consumption (AUDIT-C), should be administered annually if needed. Patients who develop AUD should be referred to suitable addiction treatment centers and supported with psychotherapy and, if needed, pharmacotherapy (e.g., naltrexone, acamprosate, disulfiram, and topiramate). Similarly, for patients showing signs of opioid or sedative use disorder, pain management and psychiatry should collaborate using a multidisciplinary approach to develop a plan.^1^^,^^6^ It is important to note that SUD is a slow-onset condition that worsens over time after BS. Therefore, follow-up with patients should continue not only during the first year but also in the long term after surgery.

Taken together, the literature suggests a clinically meaningful increase in alcohol-related risk after BS, particularly after RYGB and over longer follow-up periods. However, the magnitude and timing of this risk are not entirely consistent across studies, likely owing to differences in surgical technique, baseline substance use vulnerability, and duration of postoperative observation. Thus, while there is growing consensus that alcohol-related complications deserve routine monitoring, uncertainty remains regarding the precise mechanisms and the relative contribution of psychosocial versus biological factors.

### Personality Disorders and Mood Instability

A study examining quality of life and psychological changes in BS patients found that 34.1% of participants had personality disorders. The study reported that B cluster personality traits, including borderline personality disorder (BPD), were the most common. Mood swings, anger control problems, and fragmented self-perception characterize such personality traits in patients.^24^

A large meta-analysis reported that childhood trauma is strongly associated with BPD.^25^ Early adverse experiences are thought to contribute to the development of core features of BPD, including emotional instability and identity disturbance. These characteristics may complicate psychological adaptation after BS. Emotion dysregulation, a core dimension of borderline psychopathology, may represent an additional mechanism linking personality pathology with poorer postoperative adaptation and less favorable weight loss bariatric outcomes.^26^^,^^27^ In the context of rapid weight loss and bodily changes following surgery, some patients may experience a “who am I?” dilemma. Particularly during periods of pronounced physical transformation, patients may report an identity conflict, sometimes expressing thoughts such as “I still look the same as I did before.” Furthermore, emotional instability has been associated with both insufficient weight loss and psychosocial maladjustment in the postoperative period.^27^

Psychotherapies, such as dialectical behavior therapy (DBT), which address issues in ­self-perception after BS, can be effective for individuals with personality disorders. Dialectical behavior therapy focuses on symptoms specific to BPD, such as impulsive actions, emotional instability, anger management issues, and identity confusion. Some studies have also explored psychotherapeutic interventions in BS populations. For example, a prospective study reported that postoperative psychotherapies, including DBT and interpersonal therapy, were associated with greater weight loss and improvement in BPD symptoms among bariatric patients.^28^ However, these findings remain preliminary, and randomized controlled trials are needed to confirm the effectiveness of such interventions.

A systematic review showed that the presence of preoperative personality disorders can affect the quality of life, weight loss, behavioral compliance, and metabolic outcomes after BS.^9^ Additionally, while some studies have observed a decrease in Cluster C personality disorders after BS, Cluster B personality disorders, particularly BPD, may persist and require psychological support.^29^

European and international guidelines identify unstable and severe personality disorders as contraindications for BS.^30^ However, BPD should not be regarded as an absolute contraindication. Current clinical perspectives suggest that the main concern is not the diagnosis alone, but the presence of severe or unstable personality pathology, especially when accompanied by marked impulsivity, emotional dysregulation, recurrent self-harm, poor treatment adherence, or active psychiatric decompensation. In this context, some guidance documents and clinical frameworks consider severe personality disorder among conditions that may make a patient temporarily unsuitable for surgery or require postponement until stabilization is achieved.^26^^,^^29^^,^^30^ Therefore, a careful, individualized assessment is essential. Rather than treating BPD as a fixed contraindication, clinicians should focus on the severity, current stability, history of self-harm or suicidality, capacity for adherence, and the availability of structured psychiatric follow-up. In selected patients, psychotherapeutic interventions such as DBT may help improve emotional regulation and postoperative adjustment.

### Psychotic Disorders

Bariatric surgery is not an absolute contraindication for individuals with schizophrenia, schizoaffective disorders, or other chronic psychotic disorders. However, mental stability remains essential. European and American BS guidelines specify at least 6 months of stability, treatment adherence, and a support system as necessary prerequisites before considering surgery for individuals with psychotic disorders.^31^ In a recent retrospective study, 20 patients with schizophrenia and schizoaffective disorder using antipsychotics were compared with controls, and no significant differences in weight loss, metabolic improvement, or mortality were found after 5 years of follow-up post-BS. Additionally, there was no significant worsening in the course of psychotic disorders, and BS was found to be safe and effective.^32^

### Self-Harm and Suicide Risk

One of the most concerning psychiatric complications after BS is the increase in suicide and self-harm behaviors.^6^ The increased risk of suicide and self-harm after BS has been well documented. For example, among 16 683 patients who underwent BS and were followed up in Pennsylvania, the suicide rate was 6.6 per 10 000 (13.7 in men, 5.2 in women). This rate is significantly higher than the suicide rates in the age- and sex-matched United States population (2.4 in men, 0.7 in women). Approximately 30% of suicide attempts occur within the first 2 years after surgery, and nearly 70% occur within the first 3 years.^33^ In a meta-analysis, the suicide rate after BS was 2.7 per thousand, while the suicide/self-mutilation attempt rate was 17 per thousand. This rate was 8 times higher than the average suicide rate in countries with the highest worldwide suicide rates. Furthermore, this study found that the risk of self-mutilation and suicide attempts among patients who underwent BS was 3.8 times higher than that among age- and sex-matched controls.^34^ Some studies have reported that suicide and self-mutilation rates are higher in patients who undergo RYGB surgery than in those who undergo restrictive procedures, such as gastric banding. For example, in a study of 121 203 patients in Australia, hospital admissions related to suicide attempts declined over time after sleeve gastrectomy and gastric banding, whereas no significant decline was observed in the gastric bypass group.^35^ This indicates that the type of surgical procedure may influence the risk of suicide.

Although the mechanisms underlying the increased risk of suicide are not fully understood, several hypotheses have been proposed. For instance, psychiatric comorbidities are significant factors. Issues such as a history of trauma, depression, and substance abuse are more prevalent in patients with BS than in the general population and may play a role in suicide.^35^ Emotion dysregulation may represent an additional psychological mechanism contributing to vulnerability to suicidal ideation and self-harm, as difficulties in regulating negative emotions have been associated with impulsive and maladaptive behaviors that increase suicide risk.^36^ In particular, adverse childhood experiences have been associated with increased vulnerability to depression, anxiety, and psychological distress among individuals seeking BS.^37^ Patients with a history of childhood trauma may therefore represent a subgroup at elevated risk for adverse psychiatric outcomes following surgery.

Indeed, evidence suggests that most patients with BS who attempt suicide also have a history of mental illness before surgery. Research shows that the risk of suicide increases significantly after surgery, especially among individuals with a prior history of suicidal thoughts or attempts. Therefore, this history should be actively explored during the preoperative assessment, and if present, the patient should be closely monitored by a psychiatrist. Another factor is the feeling of “not achieving the desired result.” If patients undergo surgery with high hopes and lose weight but realize that not all of their expected life problems have been solved, they may experience serious disappointment. In some sensitive patients, this situation can lead to feelings of helplessness that may trigger suicidal ideation. Additionally, for some patients, restrictions on eating after surgery might create a psychological void in individuals who previously relied on eating to cope with their emotions, and self-harm or risky behaviors (e.g., accident proneness, impulsivity) may increase.^1^^,^^6^^,^^35^

It is crucial for the entire healthcare team to actively prevent suicide and self-harm. As part of monitoring and prevention efforts, all patients should be screened regularly for depression and suicidal thoughts after BS. Post-surgery, especially in patients with risk factors, a psychiatric evaluation is advised annually for the first 2 years, and then either yearly or every 2 years up to 5 years. If the patient reports anxiety or hopelessness beyond their control, or if family members notice changes in the patient’s behavior, a psychiatric consultation should be arranged immediately. Additionally, patients should be taught to recognize warning signs during the postoperative period. For example, symptoms such as missing follow-up appointments, not following the diet, social withdrawal, and increased alcohol use may indicate a psychological crisis.^13^ Standard crisis interventions should be implemented for patients identified as being at risk of suicide. A safe environment should be provided, and treatment, including AD treatment and psychotherapy, should be planned with close monitoring. Furthermore, support groups for individuals who have undergone BS can play a protective role by providing opportunities to share similar experiences.^6^

In conclusion, suicide and self-harm may represent serious psychiatric complications following BS. Careful preoperative risk assessment, appropriate postoperative treatment, and ongoing psychosocial support are essential for preventing these adverse outcomes.

### Causes of Psychiatric Complications

Both neurobiological and psychosocial mechanisms likely contribute to the development of psychiatric complications after BS. However, the relative contribution of these mechanisms remains uncertain, and current findings should be interpreted cautiously because many studies are observational, indirect, or based on intermediate biological markers rather than direct psychiatric endpoints.

#### Gastrointestinal Anatomy and Microbiota Changes

Bariatric surgery, particularly RYGB and sleeve gastrectomy, alters gastrointestinal anatomy and may reshape the intestinal microbiota. These changes may influence the gut-brain axis and contribute to alterations in neurotransmitter (NT) balance associated with psychiatric symptoms.^38^ Recent studies have suggested that changes, such as a decrease in microbiota diversity and an increase in inflammatory bacteria, may be associated with depression and other mental disorders.^39^

#### Hormonal and Neurochemical Changes

After RYGB, levels of hormones such as ghrelin, GLP-1, and PYY change, potentially affecting appetite regulation, reward pathways, and dopaminergic mechanisms. For instance, in the first year after RYGB, ghrelin levels decline, whereas GLP-1 and PYY levels increase, changes that are generally associated with reduced hunger and enhanced satiety.^1^ Additionally, ghrelin has been suggested to increase reward sensitivity in the dopamine system, whereas higher levels of GLP-1 and PYY may contribute to the regulation of eating behavior and have been proposed to influence vulnerability to alcohol or substance dependence.^40^

#### Vitamin–Mineral Deficiencies and Neurotransmitter Dysfunction

Thiamine, vitamin B12, folic acid, vitamin D, and magnesium deficiencies are common after BS. These deficiencies may influence NT systems, including dopamine, serotonin, and gamma-aminobutyric acid, and may contribute to the emergence of psychiatric symptoms such as depressive features, psychotic symptoms, or cognitive disturbances. In particular, deficiencies in B-group vitamins may adversely affect nervous system function, which has been associated with behavioral and mood disorders.^1^^,^^3^^,^^41^

#### Rapid Weight Loss and Psychosocial Dysfunction

Rapid weight loss may contribute to body image disturbance, psychosocial adjustment difficulties, and other lifestyle stressors. Patients may face challenges in psychosocial adaptation due to expectations following BS and distortions in body image perception.^42^

#### Preoperative Mental State

Preexisting depressive disorders, anxiety disorders, and ED may persist or exacerbate after BS.^3^

In summary, psychiatric complications after BS result from the interaction of biological, hormonal, and psychosocial factors. Recognizing these factors can improve preventive strategies. The proposed mechanisms contributing to psychiatric complications after BS are summarized in Table 4. Although several biological mechanisms have been proposed to explain psychiatric changes following BS, the current evidence remains incomplete. At present, much of the mechanistic literature should be regarded as emerging rather than definitive, particularly regarding microbiota-related pathways and alterations in the reward circuitry. Hormonal alterations, changes in gut microbiota, and modifications in reward-related neurocircuitry have all been suggested as contributing factors. However, many of these mechanisms are derived from observational or experimental studies, and their direct causal role in psychiatric outcomes after BS has not yet been fully established. Further longitudinal and mechanistic studies are needed to clarify these relationships.

#### Changes in Psychiatric Medication Treatment Due to Surgery

Malabsorptive BS procedures, particularly RYGB, significantly affect drug absorption by altering gastrointestinal anatomy. The bioavailability of many psychotropic drugs may decrease due to reasons such as accelerated gastric emptying, reduced stomach surface area, and changes in first-pass metabolism, particularly of cytochrome P450 3A enzymes. In particular, plasma levels of selective serotonin reuptake inhibitors (SSRIs) and serotonin-norepinephrine reuptake inhibitors have been reported to decrease by approximately 25% in the first few months after surgery.^43^ Weight-neutral AD drugs should be preferred. Tricyclic AD drugs should be avoided due to the risk of electrolyte imbalance. Sertraline, from the SSRI group, may be preferred because of its low risk of corrected QT interval (QTc) prolongation and its favorable safety profile regarding weight gain. Selective serotonin reuptake inhibitors with high cytochrome P450 enzyme inhibition, such as fluoxetine, fluvoxamine, and paroxetine, should be used cautiously due to potential drug interactions. Citalopram and escitalopram should also be used with caution because of the risk of QTc prolongation.^1^

The absorption of certain antipsychotic drugs, such as ziprasidone and lurasidone, which must be taken with food after BS, may vary.^1^ Since the solubility and absorption of tablet-based drugs may decrease after surgery, liquid, sublingual, or transdermal forms are preferred. Changes in drug absorption, especially for those with narrow therapeutic ranges like lithium, valproic acid, and lamotrigine, can cause serious toxicity or underdosing. Therefore, alternative formulations such as liquid, sublingual, or transdermal preparations may improve drug bioavailability and treatment adherence.^43^^,^^44^

Regular monitoring and dose adjustments are essential after BS. Clinical guidelines recommend measuring therapeutic drug levels during the first 6-12 months after surgery. Doses should be adjusted based on plasma concentrations, especially for drugs like carbamazepine and lithium. Long-acting injectable formulations, such as paliperidone palmitate, may be considered for patients who have poor medication adherence.^43^^,^^45^ In summary, treatment plans should be tailored to the individual, with dose adjustments based on blood level measurements and medication changes considered when necessary.

Pharmacokinetic considerations and clinical recommendations for psychotropic medications are summarized in Table 5.

### Treatment, Prevention, and Monitoring of Psychiatric Complications

Multidisciplinary team support is vital during the pre- and postoperative periods in BS. To prevent psychosocial complications after BS, multidisciplinary collaboration is essential. A team including surgeons, psychiatrists or psychologists, dietitians, and nurses should coordinate nutritional management, psychological support, and lifestyle interventions.^1^

Comprehensive psychoeducation should be provided before and after surgery, and the patient should be informed in advance about possible psychiatric problems and lifestyle changes. Psychotherapy applications such as cognitive behavioral therapy (CBT), mood-focused therapy, or DBT provide support, particularly in terms of body image perception, self-esteem, and coping skills.^1^ Cognitive behavioral therapy is an effective intervention, especially for ED, depression, and anxiety disorders that develop after surgery. A 2020 study found that CBT applied to patients diagnosed with BPD and major depression resulted in significant improvement in BPD and depression symptoms and increased self-esteem, with these effects persisting at the 3-month follow-up.^46^ Cognitive behavioral therapy has also been shown to provide significant benefits for ED and emotional regulation issues.^47^

When pharmacological treatment is necessary, AD drugs or anxiolytics (e.g., SSRIs such as sertraline) with a low risk of weight gain and minimal drug interactions should be preferred, and the efficacy of the drugs should be monitored regularly.^1^

Long-term follow-up after BS is also important. Evidence suggests that fewer than 50% of patients attend follow-up appointments several years after BS.^48^ Studies have shown that even if psychological symptoms improve in the first year after BS, they may increase again in some patients in later years.^33^ Therefore, regular psychiatric follow-up during the first 2-3 years after surgery and at least 1 psychological screening annually thereafter are recommended.^1^ Regular follow-up has been shown to improve patient compliance and positively influence weight loss. According to standard practices, long-term ­follow-up plans that include psychiatric care should be established starting from the first postoperative months. When needed, group therapy, telephone counseling, or tele-CBT applications can also be utilized.^30^^,^^49^

### Bariatric Surgery and Psychiatric Risks in Special Populations

#### Adolescents

Adolescent obese patients have high rates of psychiatric issues before BS. It has been shown that depression, anxiety, and ED are common among adolescents, and that mental health ­follow-up and support are critically important in the post-BS period.^50^ A recent study found that adolescent BS candidates had significantly higher rates of psychiatric diagnoses and medication use compared to a control group, both 5 years before and 10 years after surgery. Additionally, while depressive symptoms decreased in the first year after BS, the risk of substance use and psychiatric morbidity persisted in the long term.^51^ Therefore, mental health assessment becomes even more critical in adolescent patients both before and after BS. In addition to psychological support, parental counseling and multidisciplinary follow-up are essential.^52^

Bariatric surgery in adolescence also raises important ethical and developmental considerations. Adolescents represent a population with evolving autonomy and variable developmental maturity, which may influence their capacity to understand the long-term implications of surgery and the behavioral commitments required after the procedure. Current ethical literature, therefore, emphasizes careful assessment of intentionality, understanding, and the absence of coercion when evaluating adolescent candidates. In clinical practice, informed decision-making usually requires both parental consent and adolescent assent, together with a structured psychosocial evaluation to determine whether the adolescent can meaningfully participate in the decision-making process. Family dynamics are also highly relevant because caregivers play a central role in treatment adherence, nutritional supervision, and long-term follow-up. At the same time, family-related difficulties should not automatically be considered a contraindication to surgery; rather, they should be identified and addressed within a multidisciplinary framework.^53^^,^^54^

#### Elderly Individuals

In advanced-age patients who are candidates for BS, their cognitive status, multidisciplinary suitability, surgical expectations, and overall health must be carefully evaluated. The number of studies in the literature that specifically address the psychiatric outcomes of BS in elderly individuals is very limited. However, it has been suggested that individuals undergoing any surgical procedure at an advanced age are more vulnerable in terms of psychosocial adjustment due to risk factors such as malnutrition, cognitive impairment, and social isolation.^55^

#### Individuals with a History of Psychiatric Illness

Bariatric surgery may still be a significant option for obese individuals who have already been diagnosed with a psychiatric disorder. These patients, with proper follow-up after surgery, also experience significant weight loss and an improved quality of life.^2^ However, it has also been shown that BS has been associated with an increased risk of self-harm in candidates with a prior diagnosis of psychiatric disorder.^56^ Furthermore, Morgan et al^57^ reported that preexisting psychiatric disorders are among the strongest predictors of requiring psychiatric services in the postoperative period.

## Conclusion

While BS is an effective method for treating obesity, it is a multifaceted procedure that carries a risk of developing psychiatric complications after surgery. Psychiatric complications that occur after surgery may arise from the interaction of hormonal and neurochemical changes, changes in the microbiota, body image alterations due to rapid weight loss, and psychosocial stressors. This process is particularly complex in individuals with a history of psychiatric diagnosis and can lead to serious consequences such as suicidal behavior. Therefore, a thorough mental health assessment before BS, precise determination of surgical suitability, and identification of individual risk factors are critical. Long-term psychiatric follow-up is essential after BS. When necessary, pharmacological and psychotherapeutic interventions should be implemented to ensure both patient safety and sustained surgical outcomes.

Prospective studies based on long-term follow-up should be conducted to better understand and prevent psychiatric complications after BS. Furthermore, standard psychiatric follow-up protocols should be established for use in the postoperative period. Individualizing treatment plans and strengthening multidisciplinary collaboration are important for improving psychiatric outcomes and enhancing quality of life.

## Figures and Tables

**Table 1.** Types of Bariatric Surgery

Mechanism of Action	Surgical Procedure
Restrictive type	Laparoscopic adjustable gastric banding
	Laparoscopic sleeve gastrectomy
Malabsorptive type	Jejunoileal bypass
Combined restrictive and malabsorptive type	Roux-en-Y gastric bypass
	Biliopancreatic diversion with duodenal switch

**Table 2.** Psychiatric Conditions Considered Contraindications for Bariatric Surgery

Psychiatric Condition	Surgical Eligibility Status	Clinical Considerations
Active psychotic disorders	Absolute contraindication unless stabilized	Requires sustained symptom remission and treatment adherence before surgery.
Uncontrolled bipolar disorder / major depression	Relative contraindication	Surgical evaluation should follow psychiatric stabilization and suicide risk assessment.
Recent or high suicide risk	Absolute contraindication	Surgery is postponed until the patient is psychiatrically stable and has no active suicidal thoughts.
Untreated substance or alcohol use disorder	Absolute contraindication	
Untreated eating disorders (e.g., BED, bulimia)	Relative contraindication	Requires prior psychiatric intervention and symptom control.
Cognitive impairment or inability to provide informed consent	Absolute contraindication	
Persistent non-adherence to treatment	Relative contraindication	Adherence to medical, dietary, and psychiatric recommendations must be evaluated.

BED, binge eating disorder.

**Table 3.** Factors Contributing to the Recurrence or New Onset of Depression Following Bariatric Surgery

Factor Type	Description
Biological factors	
Hormonal changes	Changes in hormone levels, such as leptin and ghrelin, occur due to rapid weight loss.
Nutrient malabsorption	Decreased absorption of vitamins and minerals after surgery, particularly deficiencies in vitamin B12, vitamin D, and folate, is thought to affect mood.
Psychosocial factors*	
High expectations	People who rely heavily on weight loss to find happiness may feel empty when they do not get the results they expect.
Expectation of disappointment	Individuals who undergo surgery, believing that “all my problems are related to my weight,” may feel disappointed and hopeless if they encounter difficulties in life after losing weight.
Deficit in emotional eating behavior	For people who use food to cope emotionally, restricting this habit can lead to mood swings.
Body image disturbances	Difficulty adjusting to the new body image after rapid weight loss: Struggling to recognize oneself in the mirror, feeling that inner identity does not match physical changes, sagging skin, and more. Even with improvements in appearance, it can lead to feelings of depression.

*Psychosocial factors may be particularly prominent.

**Table 4.** Causes of Psychiatric Complications After Bariatric Surgery

Mechanism	Psychiatric Impact
Changes in gut anatomy and microbiota	Disruption of the gut-brain axis may contribute to depression and anxiety symptoms.
Hormonal adaptations (↓ Ghrelin, ↑ GLP-1, ↑ PYY)	Altered reward pathways may be associated with changes in eating habits and increased vulnerability to addictive behaviors.
Micronutrient deficiencies	Micronutrient deficiencies may contribute to neurotransmitter imbalance (e.g., dopamine, serotonin) and may be associated with mood and cognitive symptoms.
Rapid weight loss and disruption of body image	Rapid weight loss and body image changes may contribute to distorted self-perception and identity-related difficulties.
Stress related to social and interpersonal adjustment	Psychosocial stressors may be associated with relationship problems, social withdrawal, and emotional instability.
Preexisting mental health condition	Preexisting mental health conditions may increase the risk of persistence or worsening of depression, anxiety, or eating disorders.

GLP-1, glucagon-like peptide-1; PYY, peptide YY.

**Table 5.** Pharmacokinetic Changes and Clinical Guidance for Psychiatric Medications Following Bariatric Surgery

Medication Class	Pharmacokinetic Change / Risk	Potential Clinical Impact	Clinical Recommendations
Antipsychotics	Absorption of agents like lurasidone and ziprasidone may decrease due to food dependence after surgery.	Reduced absorption → Decreased efficacy	Consider depot formulations. Monitor serum levels if using oral forms.
Mood stabilizers	Serum levels of valproate, carbamazepine, and oxcarbazepine may decrease following malabsorptive procedures.	Decreased levels → Loss of efficacy	Regular therapeutic drug monitoring is recommended; dose adjustments may be necessary.
	Lamotrigine levels may fluctuate unpredictably.	Unclear → Clinical monitoring required	Adjust the dose according to clinical response.
	Lithium levels may rise significantly, and dehydration increases the risk of toxicity.	Increased levels → Toxicity risk	Monitor serum levels closely and educate patients on signs of toxicity and the importance of hydration.
Antidepressants	Tricyclic antidepressants pose a risk of electrolyte disturbance and are less commonly used.	QTc prolongation, arrhythmia risk	Avoid if possible.
	Sertraline has a favorable profile regarding QTc and weight neutrality.	Safe option → Maintains efficacy	May be used as a first-line SSRI.
	Fluoxetine, fluvoxamine, and paroxetine are potent CYP450 inhibitors.	Increased potential for drug interactions	Use with caution when combined with other medications.
	Citalopram and escitalopram pose a risk of QTc prolongation.	QTc risk ↑ → Potential cardiac side effects	ECG monitoring is recommended for patients at risk.
	Bupropion lowers the seizure threshold.	Increased risk of seizures	Not recommended for patients with a history of seizures.
	Mirtazapine is associated with weight gain.	Risk of weight gain	Use with caution in patients aiming to lose weight after surgery.

CYP450, cytochrome P450; ECG, electrocardiogram; QTc, corrected QT interval; SSRI, selective serotonin reuptake inhibitor.

## Data Availability

Data sharing is not applicable to this article, as no datasets were generated or analyzed during the current study.

## References

[1] Francois Z Rizvi A. Psychiatric complications of bariatric surgery. In: StatPearls. StatPearls Publishing; 2024. Accessed November 1, 2025. 38861640

[2] Vermeer KJ Monpellier VM Cahn W Bariatric surgery in patients with psychiatric comorbidity: significant weight loss and improvement of physical quality of life. Clin Obes. 2020;10(4):e12373. (doi: 10.1111/cob.12373) 32424972 PMC9285938

[3] Law S Dong S Zhou F Bariatric surgery and mental health outcomes: an umbrella review. Front Endocrinol (Lausanne). 2023;14:1283621. (doi: 10.3389/fendo.2023.1283621) 38027159 PMC10653334

[4] Dawes AJ Maggard-Gibbons M Maher AR Mental health conditions among patients seeking and undergoing bariatric surgery: a meta-analysis. JAMA. 2016;315(2):150 163. (doi: 10.1001/jama.2015.18118) 26757464

[5] El Alam A Fleifel M Baba H A safe path to bariatric surgery: mental health disorders in pre-operative patients. Compr Psychoneuroendocrinol. 2025;23:100300. (doi: 10.1016/j.cpnec.2025.100300) 40496710 PMC12149645

[6] Troisi A. Emergence of bariatric psychiatry as a new subspecialty. World J Psychiatry. 2022;12(1):108-116. (doi: 10.5498/wjp.v12.i1.108) PMC8783166 35111582

[7] Fried M Yumuk V Oppert JM Interdisciplinary European guidelines on metabolic and bariatric surgery. Obes Surg. 2014;24(1):42 55. (doi: 10.1007/s11695-013-1079-8) 24081459

[8] Generali I De Panfilis C. Personality traits and weight loss surgery outcome. Curr Obes Rep. 2018;7(3):227 234. (doi: 10.1007/s13679-018-0315-x) 30051313

[9] Bordignon S Aparício MJG Bertoletti J Personality characteristics and bariatric surgery outcomes: a systematic review. Trends Psychiatry Psychother. 2017;39(2):124 134. (doi: 10.1590/2237-6089-2016-0016) 28614435

[10] Alyahya RA Alnujaidi MA. Prevalence and outcomes of depression after bariatric surgery: a systematic review and meta-analysis. Cureus. 2022;14(6):e25651. (doi: 10.7759/cureus.25651) 35784972 PMC9249077

[11] By-Band-Sleeve Collaborating Group. Prevalence and short-term change in symptoms of anxiety and depression following bariatric surgery: a prospective cohort study. BMJ Open. 2024;14(1):e071231. (doi: 10.1136/bmjopen-2022-071231) PMC10773381 38171620

[12] Duarte-Guerra LS Villares JF Santo MA Relationship between psychiatric disorders and loss weight among patients underwent metabolic and bariatric surgery: a reassessment observational study after nine years. Clinics (Sao Paulo). 2024;79:100517. (doi: 10.1016/j.clinsp.2024.100517) 39432979 PMC11533480

[13] Vaishnav M Gupta S Vaishnav P. Psychiatric intervention pre- and post-bariatric surgery. Indian J Psychiatry. 2022;64(suppl 2):S473 S483. (doi: 10.4103/indianjpsychiatry.indianjpsychiatry_1_22) 35602357 PMC9122160

[14] Chao AM Wadden TA Faulconbridge LF Binge-eating disorder and the outcome of bariatric surgery in a prospective, observational study: two-year results. Obesity (Silver Spring). 2016;24(11):2327 2333. (doi: 10.1002/oby.21648) 27616677 PMC5093053

[15] Ivezaj V Barnes RD Cooper Z Loss-of-control eating after bariatric/sleeve gastrectomy surgery: similar to binge-eating disorder despite differences in quantities. Gen Hosp Psychiatry. 2018;54:25 30. (doi: 10.1016/j.genhosppsych.2018.07.002) 30056316 PMC6245943

[16] Voderholzer U Naab S Cuntz U Anorexia nervosa—an update. Nervenarzt. 2025:1 8. (doi: 10.1007/s00115-025-01820-y) 40434418

[17] Müller A Efeler S Laskowski NM Postoperative dumping syndrome, health-related quality of life, anxiety, depression, and eating disturbances: results of a longitudinal obesity surgery study. Obes Facts. 2024;17(2):201 210. (doi: 10.1159/000536602) 38320543 PMC10987184

[18] Pizato N Botelho PB Gonçalves VSS Effect of grazing behavior on weight regain post-­bariatric surgery: a systematic review. Nutrients. 2017;9(12):1322. (doi: 10.3390/nu9121322) . 29206132 PMC5748772

[19] Ivezaj V Benoit SC Davis J Changes in alcohol use after metabolic and bariatric surgery: predictors and mechanisms. Curr Psychiatry Rep. 2019;21(9):85. (doi: 10.1007/s11920-019-1070-8) 31410716 PMC7057935

[20] King WC Chen JY Mitchell JE Prevalence of alcohol use disorders before and after bariatric surgery. JAMA. 2012;307(23):2516 2525. (doi: 10.1001/jama.2012.6147) 22710289 PMC3682834

[21] Maciejewski ML Smith VA Berkowitz TSZ Long-term opioid use after bariatric surgery. Surg Obes Relat Dis. 2020;16(8):1100 1110. (doi: 10.1016/j.soard.2020.04.037) 32507657 PMC7423624

[22] King WC Chen JY Belle SH Use of prescribed opioids before and after bariatric surgery: prospective evidence from a U.S. multicenter cohort study. Surg Obes Relat Dis. 2017;13(8):1337 1346. (doi: 10.1016/j.soard.2017.04.003) 28579202 PMC5568488

[23] Chow A Neville A Kolozsvari N. Smoking in bariatric surgery: a systematic review. Surg Endosc. 2021;35(6):3047 3066. (doi: 10.1007/s00464-020-07669-3) 32524412

[24] Ramos-Bachiller B López-Gómez JJ García-Calvo S Quality of life and psychological changes in bariatric surgery: an observational study. Ann Nutr Metab. 2025;81(1):22 31. (doi: 10.1159/000540012) 39053439

[25] Porter C Palmier-Claus J Branitsky A Childhood adversity and borderline personality disorder: a meta-analysis. Acta Psychiatr Scand. 2020;141(1):6 20. (doi: 10.1111/acps.13118) 31630389

[26] Barbuti M D’Alessandro G Weiss F The impact of negative emotional dysregulation on the outcome of bariatric surgery in patients with severe obesity: an observational one-year follow-up study. J Clin Med. 2024;13(17):5158. (doi: 10.3390/jcm13175158) 39274371 PMC11395976

[27] Barbuti M Carignani G Weiss F Eating disorders and emotional dysregulation are associated with insufficient weight loss after bariatric surgery: a 1-year observational follow-up study. Eat Weight Disord. 2023;28(1):49. (doi: 10.1007/s40519-023-01574-z) 37266717 PMC10237075

[28] Gallé F Cirella A Salzano AM Analyzing the effects of psychotherapy on weight loss after laparoscopic gastric bypass or laparoscopic adjustable gastric banding in patients with borderline personality disorder: a prospective study. Scand J Surg. 2017;106(4):299 304. (doi: 10.1177/1457496917701670) 28376683

[29] Peterhänsel C Wagner B Dietrich A Obesity and co-morbid psychiatric disorders as contraindications for bariatric surgery?-A case study. Int J Surg Case Rep. 2014;5(12):1268 1270. (doi: 10.1016/j.ijscr.2014.11.023) 25460490 PMC4275787

[30] Fausta M Claudio C Mario M Psychological and psychiatric standardized procedures for metabolic bariatric surgery: a clinical practice model for mental health providers. Updates Surg. 2025;77(7):1951 1966. (doi: 10.1007/s13304-024-02053-5) 39644446

[31] Mechanick JI Youdim A Jones DB Clinical practice guidelines for the perioperative nutritional, metabolic, and nonsurgical support of the bariatric surgery patient—2013 update: cosponsored by American Association of Clinical Endocrinologists, the Obesity Society, and American Society for Metabolic & Bariatric Surgery. Surg Obes Relat Dis. 2013;9(2):159 191. (doi: 10.1016/j.soard.2012.12.010) 23537696

[32] Miñambres I Rubio-Herrera MÁ Nicolau J Outcomes of bariatric surgery in patients with schizophrenia. Nutrients. 2024;16(15):2487. (doi: 10.3390/nu16152487) 39125367 PMC11313780

[33] Tindle HA Omalu B Courcoulas A Risk of suicide after long-term follow-up from bariatric surgery. Am J Med. 2010;123(11):1036 1042. (doi: 10.1016/j.amjmed.2010.06.016) 20843498 PMC4296730

[34] Castaneda D Popov VB Wander P Risk of suicide and self-harm is increased after bariatric surgery-a systematic review and meta-analysis. Obes Surg. 2019;29(1):322 333. (doi: 10.1007/s11695-018-3493-4) 30343409

[35] Sumithran P Roberts L Caterson ID Incidence of adverse mental health outcomes after sleeve gastrectomy compared with gastric bypass and restrictive bariatric procedures: a retrospective cohort study. Obesity (Silver Spring). 2023;31(7):1913 1923. (doi: 10.1002/oby.23757) 37368518 PMC10946809

[36] Rogante E Cifrodelli M Sarubbi S The role of emotion dysregulation in understanding suicide risk: a systematic review of the literature. Healthcare (Basel). 2024;12(2):169. (doi: 10.3390/healthcare12020169) 38255058 PMC10815449

[37] Tabone JK Cox S Aylward L Addressing adverse childhood experiences and psychological symptoms among bariatric patients. J Child Adolesc Trauma. 2023;16(2):321 327. (doi: 10.1007/s40653-022-00491-0) 37234836 PMC10205957

[38] Almheiri RT Hajjar B Alkhaaldi SMI Beyond weight loss: exploring the neurological ramifications of altered gut microbiota post-bariatric surgery. J Transl Med. 2025;23(1):223. (doi: 10.1186/s12967-025-06201-2) 39994634 PMC11852891

[39] Brown RM Guerrero-Hreins E Brown WA Potential gut–brain mechanisms behind adverse mental health outcomes of bariatric surgery. Nat Rev Endocrinol. 2021;17(9):549 559. (doi: 10.1038/s41574-021-00520-2) 34262156

[40] Schulz C Vezzani C Kroemer NB. How gut hormones shape reward: a systematic review of the role of ghrelin and GLP-1 in human fMRI. Physiol Behav. 2023;263:114111. (doi: 10.1016/j.physbeh.2023.114111) 36740132

[41] Benítez T Caixàs A Rebasa P Psychopathological profile before and after bariatric surgery. Sci Rep. 2023;13(1):16172. (doi: 10.1038/s41598-023-43170-2) 37758783 PMC10533840

[42] Johnston L Jackson K Hilton C The forgotten patient: a psychological perspective on the implementation of bariatric surgery guidelines. Obes Sci Pract. 2023;9(5):538 547. (doi: 10.1002/osp4.670) 37810523 PMC10551119

[43] Lau C van Kesteren C Smeenk R Impact of bariatric surgery in the short and long term: a need for time-dependent dosing of drugs. Obes Surg. 2023;33(10):3266 3302. (doi: 10.1007/s11695-023-06770-5) 37594672 PMC10514130

[44] Lee H Kameg B Palmer J Pharmacokinetic changes in medications after bariatric surgery: a scoping review. J Nurse Pract. 2024;20(7):105043. (doi: 10.1016/j.nurpra.2024.105043) 40661342 PMC12258421

[45] Konstantinidou SK Argyrakopoulou G Dalamaga M The effects of bariatric surgery on pharmacokinetics of drugs: a review of current evidence. Curr Nutr Rep. 2023;12(4):695 708. (doi: 10.1007/s13668-023-00498-5) 37857987 PMC10766679

[46] Rudolph A Hilbert A. Cognitive-behavioral therapy for postbariatric surgery patients with mental disorders: a pilot study. Front Psychiatry. 2020;11:14. (doi: 10.3389/fpsyt.2020.00014) 32116836 PMC7028699

[47] Price C Fraser K Bartel S Screening and treating disordered eating in weight loss surgery: a rapid review of current practices and future directions. Obesities. 2025;5(2):19. (doi: 10.3390/obesities5020019)

[48] Wagner J Roll M Lautenbach A Patients’ expectations and perspectives on follow-up care after bariatric surgery in Germany. Obes Surg. 2025;35(6):2174 2184. (doi: 10.1007/s11695-025-07890-w) 40304842 PMC12130090

[49] Budny A Janczy A Szymanski M Long-term follow-up after bariatric surgery: key to successful outcomes in obesity management. Nutrients. 2024;16(24):4399. (doi: 10.3390/nu16244399) . 39771020 PMC11679841

[50] Srivastava G. Bariatric surgery in adolescents-a vital treatment option. Transl Pediatr. 2024;13(8):1287 1289. (doi: 10.21037/tp-24-160) 39263288 PMC11384432

[51] Bruze G Järvholm K Norrbäck M Mental health from 5 years before to 10 years after bariatric surgery in adolescents with severe obesity: a Swedish nationwide cohort study with matched population controls. Lancet Child Adolesc Health. 2024;8(2):135 146. (doi: 10.1016/S2352-4642(23)00311-5) 38159575

[52] Herget S Rudolph A Hilbert A Psychosocial status and mental health in adolescents before and after bariatric surgery: a systematic literature review. Obes Facts. 2014;7(4):233 245. (doi: 10.1159/000365793) 25059420 PMC5644788

[53] Martinelli V Singh S Politi P Ethics of bariatric surgery in adolescence and its implications for clinical practice. Int J Environ Res Public Health. 2023;20(2):1232. (doi: 10.3390/ijerph20021232) 36673981 PMC9859476

[54] Pratt JSA Browne A Browne NT ASMBS pediatric metabolic and bariatric surgery guidelines, 2018. Surg Obes Relat Dis. 2018;14(7):882 901. (doi: 10.1016/j.soard.2018.03.019) 30077361 PMC6097871

[55] Bokhari SFH Waseem AB Raza H Impact of psychiatric disorders on surgical outcomes: a comprehensive review of preoperative screening and interventions. World J Surg Proced. 2025;15(1). (doi: 10.5412/wjsp.v15.i1.104178)

[56] Goueslard K Jollant F Petit JM Self-harm hospitalization following bariatric surgery in adolescents and young adults. Clin Nutr. 2022;41(1):238 245. (doi: 10.1016/j.clnu.2021.11.034) 34915275

[57] Morgan DJR Ho KM Platell C. Incidence and determinants of mental health service use after bariatric surgery. JAMA Psychiatry. 2020;77(1):60 67. (doi: 10.1001/jamapsychiatry.2019.2741) 31553420 PMC6763981

